# Supplementary material for: EZH2-Mediated H3K27 Trimethylation in the Liver of Mice Is an Early Epigenetic Event Induced by High-Fat Diet Exposure
Source: Nutrients. 2024 Sep 26;16(19):3260. doi: 10.3390/nu16193260 (PMC11479199; doi:10.3390/nu16193260)
Supplement: Supplementary file 1 [file nutrients-16-03260-s001.zip › nutrients-3158124-supplementary.pdf]

A

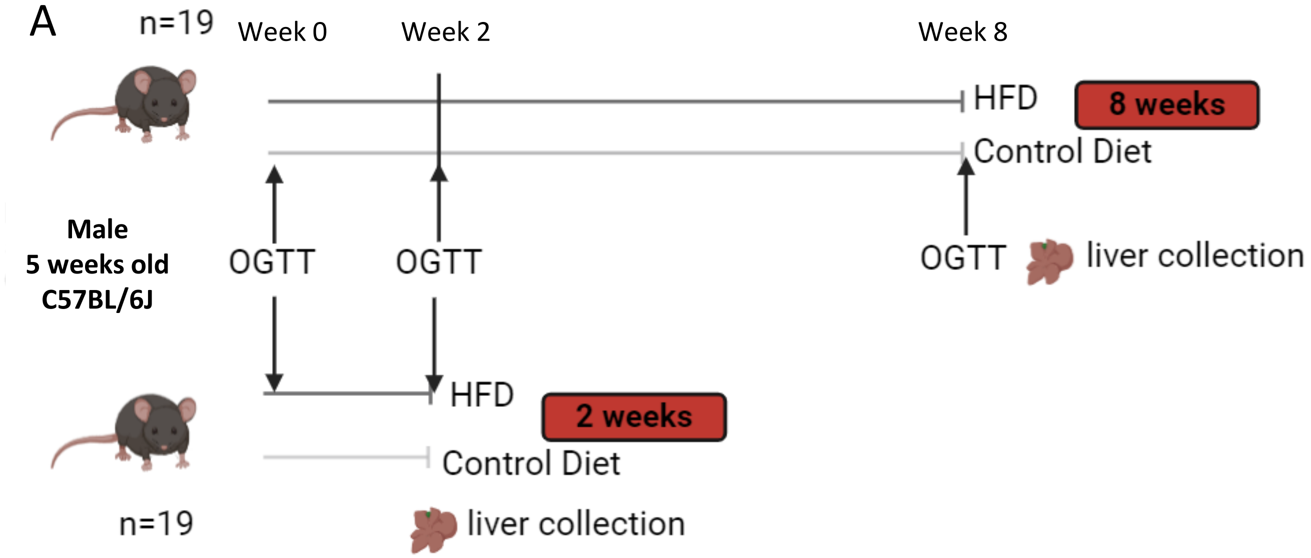

B

Control Diet	Gm%	Kcal%
Carbohydrate	67.3	70%
Protein	19.2	20%
Fat	4.3	10%
**Ingredient**	**Gm**	**Kacl**
Casein, 80 Mesh	200	800
L-Cystein	3	12
Corn Starch	315	1260
Maltodextrine 10	35	140
Sucrose	350	1400
Cellulose, BW200	50	0
Soybean Oil	25	225
Lard	20	180
Mineral Mix S10026	10	0
DiCalcium Phosphate	13	0
Calcium Carbonate	5.5	0
Potassium Citrate, 1 H2O	16.5	0
Vitamin Mix V10001	10	40
Choline Bitartrate	2	0
FD&C Yellow Dye #5	0.05	0

High Fat Diet	Gm%	Kcal%
Carbohydrate	26.3	20%
Protein	26.2	20%
Fat	34.9	60%
**Ingredient**	**Gm**	**Kacl**
Casein, 80 Mesh	200	800
L-Cystein	3	12
Corn Starch	0	0
Maltodextrine 10	125	500
Sucrose	68.8	275.2
Cellulose, BW200	50	0
Soybean Oil	25	225
Lard	245	2205
Mineral Mix S10026	10	0
DiCalcium Phosphate	13	0
Calcium Carbonate	5.5	0
Potassium Citrate, 1 H2O	16.5	0
Vitamin Mix V10001	10	40
Choline Bitartrate	2	0
FD&C Yellow Dye #5	0.05	0

Figure S1 Panel A graphical representation of the experimental *in vivo* timeline of diet supply, oral glucose tolerance tests (OGTT) and livers collection. Panel B Composition of Control and High Fat Diet.

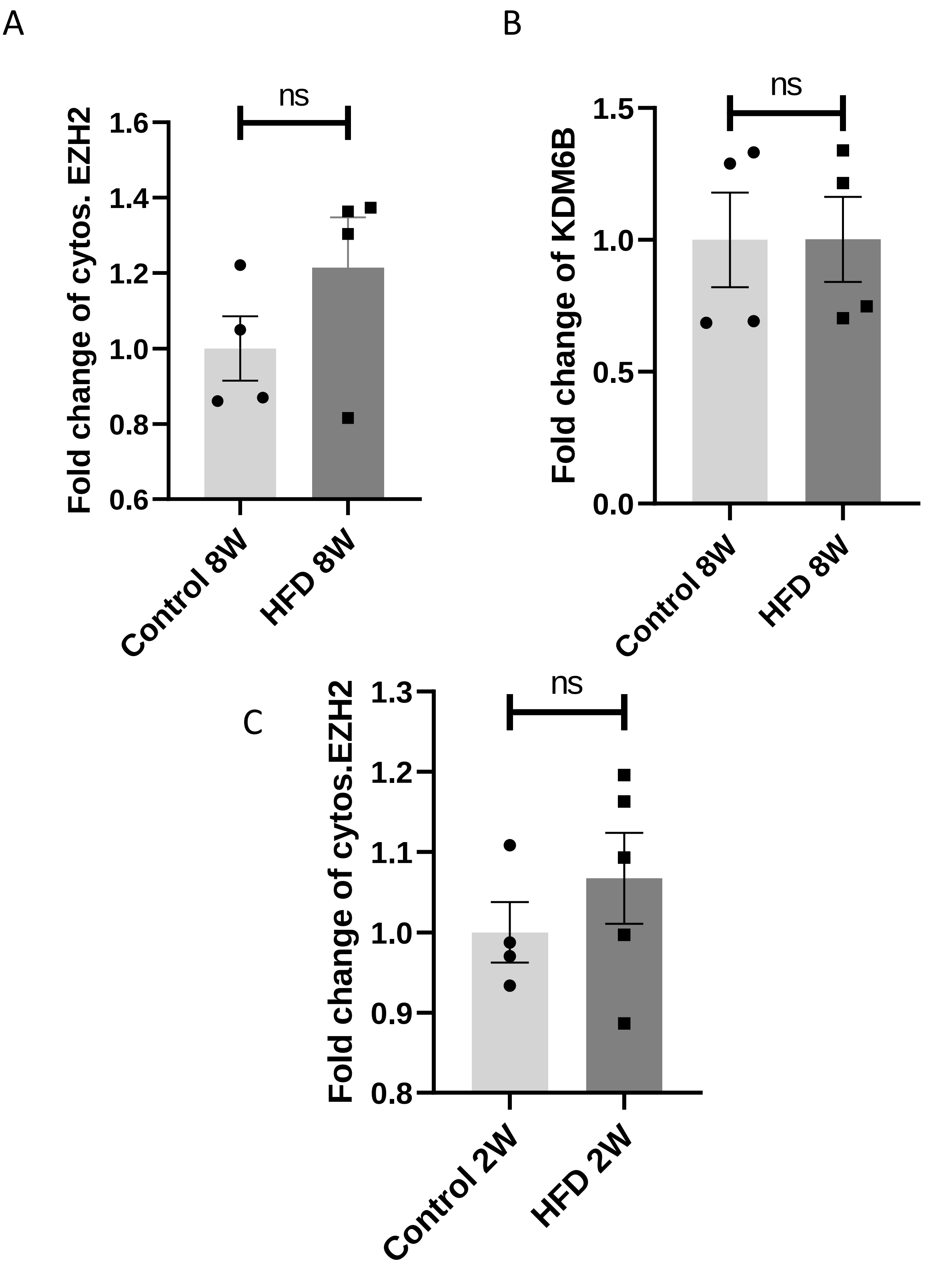

Figure S2 A. Fold change of cytosolic expression of EZH2 after 8 weeks of Control or HFD. B. Fold change of KDM6B after 8 weeks of Control or HFD. C. Fold change of cytosolic concentration of EZH2 after 2 weeks of Control or HFD
